# Stigma trajectories among people living with HIV (PLHIV) embarking on a life time journey with antiretroviral drugs in Jinja, Uganda

**DOI:** 10.1186/1471-2458-13-804

**Published:** 2013-09-05

**Authors:** Martin Mbonye, Sarah Nakamanya, Josephine Birungi, Rachel King, Janet Seeley, Shabbar Jaffar

**Affiliations:** 1 MRC/UVRI Uganda Research Unit on AIDS, P.O. Box 49, Entebbe, Uganda; 2 The AIDS Support Organisation, Kampala, Uganda; 3 University of California, San Francisco, CA, USA; 4 School of International Development, University of East Anglia, Norwich, NR4 7JT, UK; 5 London School of Hygiene and Tropical Medicine, Keppel Street, London WC1E 7HT, UK

**Keywords:** Stigma, Antiretroviral therapy, Disclosure, Uganda, Side effects, Sero-sorting

## Abstract

**Background:**

Stigma is a barrier to HIV prevention and treatment. There is a limited understanding of the types of stigma facing people living with HIV (PLHIV) on antiretroviral therapy (ART). We describe the stigma trajectories of PLHIV over a 5-year period from the time they started ART.

**Methods:**

Longitudinal qualitative in-depth interviews were conducted with 41 members of The AIDS Support Organisation (TASO) from 2005 to 2008 in Jinja, Uganda, who were part of a pragmatic cluster-randomised trial comparing two different modes of ART delivery (facility and home). Participants were stratified by gender, ART delivery arm and HIV stage (early or advanced) and interviewed at enrolment on to ART and then after 3, 6, 18 and 30 months. Interviews focused on stigma and ART experiences. In 2011, follow-up interviews were conducted with 24 of the participants who could be traced. Transcribed texts were translated, coded and analyzed thematically.

**Results:**

Stigma was reported to be very high prior to starting ART, explained by visible signs of long-term illnesses and experiences of discrimination and abuse. Early coping strategies included: withdrawal from public life, leaving work due to ill health and moving in with relatives. Starting ART led to a steady decline in stigma and allowed the participants to take control of their illness and manage their social lives. Better health led to resumption of work and having sex but led to reduced disclosure to employers, colleagues and new sexual partners. Some participants mentioned sero-sorting in order to avoid questions around HIV sero-status. A rise in stigma levels during the 18 and 30 month interviews may be correlated with decreased disclosure. By 2011, ART-related stigma was even more pronounced particularly among those who had started new sexual relationships, gained employment and those who had bodily signs from ART side-effects.

**Conclusion:**

This study has shown that while ART comes with health benefits which help individuals to get rid of previously stigmatising visible signs, an increase in stigma may be noticed after about five years on ART, leading to reduced disclosure. ART adherence counselling should reflect changing causes and manifestations of stigma over time.

## Background

Stigma is a major barrier in the prevention and treatment of HIV [1,2]. Stigma associated with HIV and AIDS is a complex concept that has been described as the ‘third epidemic’ [3]. Goffman (1963) defined stigma as “an attribute that is deeply discrediting,” and that reduces the bearer “from a whole and usual person to a tainted, discounted one”. It refers to “bodily signs designed to expose something unusual and bad about the moral status of the signifier” [4]. UNAIDS defines both stigma and discrimination as a “process of devaluation” of people either living with or associated with HIV and AIDS. Discrimination is associated with stigma and refers to the unfair and unjust treatment of an individual based on his or her real or perceived HIV status [5-7]. While Castro and Farmer [8] argue that better access to treatment has the potential to eliminate stigma, there are still concerns that even with the roll out of antiretroviral therapy (ART) in many Sub-Saharan African settings, being infected with HIV is still feared and morally judged, pointing to the persistence of stigma [9,10].

HIV infection has historically been associated with immorality and despite advances in knowledge about HIV and treatment options, the culture of blame remains relatively strong in many Sub-Sahara African settings [11]. The fear that surrounds being infected comes from the realization that one may be found out and judged if others know their HIV-status [12,13]. A South African study on stigma experiences of people on ART, concluded that the body of a person living with HIV is a highly stigmatized one and this places a heavy burden on PLHIV [14]. Another study described the stigma trajectories that PLHIV go through at different stages after the confirmation of their status, with the need to conceal being a key strategy especially during the asymptomatic phase [15]. In order to manage the concealment, withdrawal from social activities is a favoured approach [16]. Withdrawing from social activities as a strategy against felt and enacted stigma has already been documented in a Ugandan population [17-19].

Social stigma contributes to the low uptake of prevention and treatment services for HIV/AIDS in many African settings; this is closely linked to reluctance to disclose HIV sero-status for fear of being labelled or blamed [20]. Yet the disclosure of HIV status is commonly encouraged as part of positive living and to prevent the further transmission of HIV, with some studies reporting benefits from public disclosure [21]. While ART allows people to live a ‘normal’ life, the approach to sexual relationships tends to become difficult to negotiate within a context of a community that is aware of one’s status. One strategy that is increasing in popularity among PLHIV is sero-sorting where HIV-positive partners get together to avoid the blame and guilt associated with possibly infecting someone else [22-24]. However, some have argued that sero-sorting needs to be approached with caution since it may not be that effective especially among those who have not confirmed HIV status [25].

With the wide scale ART roll out and better management of HIV in much of Sub-Saharan Africa, the fear of developing AIDS has now decreased [20]. However, there are continued challenges of the low uptake of voluntary counselling and testing and the late presentation of men, for example, for treatment, which may point to an underlying epidemic of stigma which continues to persist [26-28].

In this paper we report on the stigma experiences of a sample of adults attending The AIDS Support Organisation (TASO) Jinja branch clinic after confirmation of HIV infection through to starting and staying on ART for a period of about three years (2005–2008). We also report on the experiences of the participants who were followed up three years later in 2011.

## Methods

This was a longitudinal qualitative follow up study with participants drawn from members of TASO in Jinja district, Eastern Uganda. TASO has over 11 centres located throughout Uganda that offer counselling, medical care and other social services to over 90,000 HIV infected persons and household members [29]. This study was part of a trial which is described briefly below.

### The Jinja trial

We conducted a nested qualitative study in a cluster-randomized trial which compared ART delivery at home to that of clients receiving drugs from the TASO Jinja centre. The trial has been described previously [30]. In brief, 44 geographical areas in nine strata, defined according to the ratio of urban and rural participants plus distance from the clinic, were randomised to the home-based delivery arm, using trained lay workers or standard facility-based care by drawing sealed cards from a box. The trial was integrated into normal service delivery. In accordance with guidelines at the time, all patients with WHO stage IV or late stage III disease or CD4-cell counts fewer than 200 cells per μL who started antiretroviral therapy between February 15, 2005, and December 19, 2006, were eligible. Follow-up continued until January 31, 2009. The primary endpoint was virological failure, defined as HIV plasma RNA more than 500 copies per mL after 6 months of treatment. The trial showed that the home-based HIV-care strategy was approximately equivalent to a standard clinic-based strategy, and therefore could enable improved and equitable access to HIV treatment, especially in areas with poor infrastructure and access to clinic care [30].

### The social science longitudinal qualitative sub study (2005–2008)

The qualitative study was conducted within a radius of 40 kilometres instead of the 75 kilometre catchment area of the TASO Jinja clinic. This was deliberately done to manage transport costs during the follow up period. The main economic activity of the clients that TASO serves in this area is small-scale farming. There is widespread poverty in Jinja district [17].

### Qualitative sample selection

We selected 41 participants from the main trial (N = 1453). We planned to recruit equal numbers of men and women but the death of one male participant during the enrolment phase, led to his being replaced and so instead of 40 people we recruited 41. Participants were further stratified by delivery arm, with the home delivery arm having 21 participants while the facility delivery arm had 20 participants. Further stratification was based on immunological or clinical stage, with those who had a CD4 count of between 150 and 200 or having WHO stage 1 or 2 being compared to those who had a starting CD4 of 100 or below and or with WHO stage 3 or 4. This was done to compare adherence levels in the first few months on ART between the two groups. Within each group, participants were selected consecutively. Data collection was done at different time periods to capture changes on ART over time. In the preparatory stages, we conducted two focus group discussions with ART clients and providers which formed the basis of the in-depth interview topic guides. These were pre-tested by experienced and trained interviewers.

### The longitudinal in-depth interviews

The first in-depth interview, which was conducted at the TASO clinic in 2005, was the enrolment interview and the main purpose of this interview was to capture expectations and hopes about ART while also exploring illness experiences in the past. We also used this interview to build rapport. At this interview, participants were asked for permission to visit them in their homes for subsequent interviews which they all consented to. These interviews were conducted in a private room within the social science offices located in a separate building from the TASO clinic. This helped create a free environment for participants to express themselves.

We then conducted interviews as well as home observations at 3, 6, 18 and 30 months on ART. At enrolment we interviewed 41 participants (21 male and 20 female). At month three we managed to conduct 30 interviews. Of these, 16 were men because four men could not be accessed at the time, while one had died earlier. The other 14 were women, five women could not be reached and one had died. During these interviews we sought to capture the home context, noting the facilitators and barriers to adherence as well as the early experience of participants. We also looked at new developments and how these were affecting stigma. At month 6 we conducted 34 interviews of which 20 were with men (one man had died) and 14 were with women. By this time two further women had died and three others could not be reached. These interviews followed the same themes as the ones conducted at month three. At 18 months we interviewed 18 men (three had died by then) and 17 women (three had died by then). At this time we focused on longer term experiences with ART and any experiences of stigma now that many had resumed public life, including returning to work. For those who had started new sexual relationships, we asked about factors influencing partner choice, disclosure and reproductive health issues. At 30 months we interviewed 33 participants of which 17 were men (three had died and one refused) while the women were 16 (four had died). These interviews covered long term experiences with ART and stigma, as well as summarising experience over the study period. We also paid attention to the emerging narratives of changing patterns in stigma. At the end of these interviews, we asked the participants to assess themselves using percentages about how they felt stigma may have changed over the two and a half year period since starting ART.

Home observations were guided by a checklist which included important cues such as: neighbourhood environment, medicine storage, interaction of participant with external environment, household support structure and information that could facilitate understanding of barriers or enablers for adherence. One male and two female interviewers, recruited participants at enrolment and each interviewer stayed with the same participants throughout the study to ensure continuity and allow for building and strengthening of the rapport with the participants.

Interviews were tape recorded with permission and predominantly conducted in Luganda language with only two participants interviewed in English. These interviews were transcribed and translated later with care being taken to use identifiers rather than names to maintain confidentiality. We developed an identifying system whereby the first letter represented gender (Male - M or Female - F), the next letter represented the ART delivery arm (F-for facility and H for Home delivery), and the third letter represented the clinical/Immunological stage (L for low CD4 count below 100 and H for high CD4 over 150). The final digit was a number which represented one up to five participants in a particular stratum. In one stratum this number reached six because of the participant recruited to replace the participant who passed away during the enrolment phase. The quotations used in the manuscript will adopt these codes as identifiers. Interviews lasted for about one hour although the 30 month interviews were slightly longer.

### Focus group discussions (FGDs) with TASO staff (after 30-month interviews)

We also conducted two focus group discussions, one with TASO staff engaged in service delivery at the clinic (this comprised of trained counsellors) and another with the lay field officers who took drugs to participants in their homes. The focus group discussion with facility based professional counsellors was conducted at the TASO Jinja offices in a private location and it included 13 participants, four of whom were men while the rest were women, reflecting the gender distribution of the staffing in the counselling department. On the other hand, the group discussion with lay workers who delivered drugs to the homes of TASO clients comprised of 12 men and one woman. This also reflected the ratio of men to women in this department. Both interviews lasted for about one hour and a half. These were conducted after the 30 months interviews to capture provider perspectives on adherence and the role of the provider in facilitating it.

### Longer term follow up interviews (2011)

We did a further follow-up of this social science cohort in 2011 to capture more recent trends. Informal semi-structured in-depth interviews using an interview guide were conducted with 24 of the 34 participants who we were able to re-contact. Key themes explored included: participants’ experience with ART adherence over the years, perceptions about stigma, changes in lives and livelihoods and how this fitted into their routine. These interviews were conducted at the homes of the participants. Some participants had shifted to places which were distant and this meant we could not reach them, while others had died in the period between 2008 and this follow up. The relatives of those who had died explained what they thought the cause of death had been (fever, alcohol, unknown causes, for example). By 2011 seven of the 41 participants had died.

### Data analysis

Thematic coding was aided by the use of NVivo 8 software. Analysis focused on how stigma was being encountered at both personal and community levels. We used a chronological approach to map out the changes in stigma over time noting how the different time points after initiation of ART represented new opportunities and challenges. Two junior social scientists and one senior social scientist participated in the initial stages of developing the code book. This team read the same five scripts from each interview round and identified themes that related to stigma or experiences which might depict stigma. A group meeting was then held which looked at themes from both the topic guide and any that might have emerged inductively. In case of disagreement, a team meeting was held and a consensus reached. This process continued until a code book that captured stigma trajectories over the two studies was obtained.

A master NVivo 8 project was then developed and this was controlled by one of the senior social scientists. The analysis team (which also included two senior social scientists) started the coding process with weekly meetings being held to ensure inter coder reliability. At the end of the process, the three different projects were merged into the master copy. We plotted individual experiences based on what the interviewers observed over time and the narratives of the participants in order to gain a nuanced understanding of their experiences with stigma and ART. The final themes are presented in the results and illustrative quotations are provided from different time points to show changing patterns. Thematic analysis, using themes drawn from the 2005–2008 study, was also used for the analysis of the 2011 data.

The study was approved by the Uganda Virus Research Institute Science and Ethics Committee, the Uganda National Council for Science and Technology as well as the ethics boards of the US Centers for Disease Control and Prevention, and London School of Hygiene and Tropical Medicine. Written informed consent was given before all the interviews while verbal consent was secured for the focus group discussions. Participants were offered a snack and a soft drink at enrolment since interviews were held at the clinic. In subsequent interviews at home (3, 6, and 18 months and in 2011) we offered them a bar of laundry soap. For the 30-month interview we gave a large plastic washing basin as a token of appreciation.

## Results

### Sample characteristics

The age range of the participants at enrolment was between 22 and 62 years with a median age of 35 for women and 40 years for men. A number of participants who did not have a sexual partner at enrolment reported having secured one in later months and also during recent follow up interviews in 2011. Many of the women reported being widowed at enrolment although some of these had initiated new partnerships by the thirtieth month. Most were Christians (Catholics, Anglicans and Pentecostals) with only four Muslims. Participants were from very poor social economic backgrounds and depended on close family and friends for a living especially in the first year on ART. However, within the first year of initiating treatment many resumed work and became independent once they had regained their strength. Throughout the course of the research, most people performed low skilled work like small-scale farming around homes and offering similar services for a fee in the neighbourhood, selling retail goods near homes or working in nearby fishing villages. A few remained jobless and dependent on support systems, while others said they had become housewives after securing supportive new sexual partners. Below is a vignette that looks at the stigma experience of one participant:

### Stigma trajectory of a female participant

FFL3 was a Muslim woman of 30 years who had four children by the time of the enrolment interview. She had separated from her partner. She first suspected possible HIV infection when she was told by a friend who had met her former partner and reported that the partner had lost a lot of weight and looked very frail. Her suspicions grew stronger when she saw the former partner’s condition and she also started developing illnesses which worsened rapidly, until she had to be admitted to hospital. It was then that she discovered that she too was infected with HIV. Her reaction to this was fear that she was going to die and leave her children without anyone to take care of them. She also felt shame and guilt for bringing HIV to her family and as a result decided to withdraw from the public eye for fear of being blamed for having HIV. After a few months on ART she noticed a dramatic improvement in her health and regained her former weight. She was back to her former beauty, by her own admission, and was no longer afraid of mixing with other people. She opened up a retail shop and a vegetable shop near her residence. This allowed her to continue taking her drugs on time and she was really happy with how things were going. With time she started reconsidering her stance of never resuming sexual relationships but was worried about the reaction of those in her community who had known about her illness. She eventually met a man who was a night watchman but who resided far away from her village. She did not tell him about her status and the fact that she was on ART. He also never asked about her history but wanted to have a child. Condoms were only used for a short while and soon she noticed that she was pregnant.

At a later stage we were informed by her medicine companion that she was not well (the role of a medicine companion is described elsewhere [31]). Things began to change. Apparently she was advised by a friend attending the TASO clinic that she needed to abort as a pregnancy was not good for someone of her status. She also feared the possible negative reaction from people who would see that she was now sexually active. She attempted an abortion from a fraudulent doctor on the recommendation of a friend who had successfully had an abortion from this person. She soon developed complications leading to her hospitalization and eventual death.

This story represents a series of occurrences that impacted on one woman’s life while taking ART. Her fear of possible negative reactions to resuming sex, which many shared in this study, led her to look outside her community for a partner and reproductive demands soon followed leading to a pregnancy. We see how a very positive outcome (starting ART and getting rid of stigmatizing signs) led to a fatal action based on how she interpreted her options at the time.

### Coping with HIV infection

In order to appreciate the changes in the lives of the participants it is important to look at how they first encountered the diagnosis as well as their own perception of HIV/AIDS before they got the infection.

Prior to finding out about her status, participant FFH3 confessed that she could not imagine herself with the infection. She said:

‘*Before I learnt that I was infected, if one told me that I had HIV/AIDS, we could even quarrel, “did you infect me, I would ask such a person?”.*

But eventually she experienced continuous illnesses which she tried to ignore but their persistence forced her to confront the possibility of HIV infection. She had earlier lost a husband to what was then described as cancer, which in itself was one way of disguising AIDS-related disease in the community [29]. She said:

*I realized that I was sick and was always having fever and I said that: “mother, could it be that this man died of HIV/AIDS?”* [She continued to narrate about her late husband] *He never told me that he had HIV. We* [meaning her late husband] *were even admitted to Jinja hospital but he told me he was suffering from cancer.*

To protect the reputations of the deceased, the cause of death was often concealed which raised the levels of suspicion and fear. A number of participants had moved in with supportive relatives when they were weak and unhealthy following prolonged illnesses. This environment of shame and blame was best managed carefully in order to minimize the assumed damage to their reputation. As a result each individual seemed to tap into personal reserves to cope with real and assumed stigma. At the 30 month-interviews, participants made self-assessments about how they rated themselves in terms of stigma. Nine women reported that they felt stigma at levels between 90 and 100% at enrolment. Explaining, FFH3 felt very shy and ashamed, in her own words she said: *I even feared going to TASO. I used to fear a lot and someone even told me that they give you drugs to kill you if you went to TASO.* FFH4 was more worried about people finding out through her daily drug taking: *I had much stigma I wondered what people would say when they saw me swallowing the tablets.* However, she commented: *No one rejected me at all. That was within me, myself*.

From such narratives, we see the source of stigma being multiple and encompassing the infected person, their immediate and external environment as well as the social support organisation and yet they had to belong to the support organisation to manage their progressing disease.

### How initiating ART affected stigma

Having initiated ART, the participants, especially the women, anticipated great changes in their health. Many had witnessed the positive effects of Cotrimoxazole prophylaxis and were anticipating even better changes from ART, having heard and seen the changes in other TASO members who were already taking the medication. They said that they hoped to avoid the side effects that could potentially reverse the health gains. It seemed from the perspective of many that the aim was to keep visible body signs at bay with the hope that initiating ART would help achieve this. Indeed in the first few months this hope had turned into reality and almost all the participants were fully satisfied with how their lives had been transformed. One participant, however, expressed concerns that the signs of infection were not disappearing as quickly as she had anticipated:

*…The skin was bothering me but now I can see that it is improving. It will improve, but it seems here* [pointing at part of the skin with a rash] *I was bitten by mosquitoes. I fear people who see me; they may ask themselves that what is wrong with her?* (FFH4, 30-month interview)

### Continuing side effects and stigma

As the longitudinal qualitative study came to an end in 2008, we began hearing about changes in the way people felt about themselves. Then when we conducted the follow up interviews in 2011 we observed that for some participants ART was no longer the magic bullet that was taking care of stigmatizing signs on their bodies. In fact these people had started connecting the new forms of strange bodily signs with ART. This had the potential to bring back strong feelings of shame. The signs reported included: loss of facial fat and darkening of the skin. Both men and women reported these signs but the women seemed particularly concerned. One participant who was experiencing these changes presented their feelings in the following illustration:

*They told us that if you take these drugs, you don’t get other sicknesses but now you see how my eye looks, I thought that if I take the drugs for a long time all the sicknesses would go and I remain with only the virus*. (FHL1 in 2011)

The changing stigma trajectories associated with bodily signs for one participant, MHH3, are illustrated in the extracts below:

At 3 months he had no problems with home visits by TASO, having been randomized to receive drugs at home:

…I’m very thankful that I am here in Uganda, in Jinja and I am even able to receive my drugs from home.

At 6 months on ART, the positive feelings continued:

…there has been a great change because since I started taking the drugs, I have never fallen seriously sick. I sometimes even forget I have HIV.

A similar response was reported throughout the rest of the follow up period up to 30 months on ART, but there was a marked change in attitude when re-contacted in 2011. The participant had by then experienced side-effects and was not as happy with the bodily changes he was experiencing:

*You see my mouth has changed its shape and even my face is pale with sunken eyes. Even though I try to add some Vaseline on my face it doesn’t change. People can think that I don’t bathe properly according to the way my skin looks. As a human being, I feel bad but there is nothing to do about it. I don’t mind if one talks about me and I can’t quarrel with him or her but in my mind it hurts me*. (MHH3 in 2011)

### Resumption of public life

Not all participants reported poor results from ART and in fact the majority were very satisfied, and felt that they had fully reintegrated into society and started interacting with other people without fear of being isolated. As a result these people had started to work again, thus gaining greater independence. However new challenges were registered for those who were employed or who worked within a public setting as well as some who had been randomized to receive drugs from the home based arm. The labelled vehicles and motorcycles were by 2008, becoming a source of discomfort to the extent that by the 30-month interview, strategies and arrangements were being negotiated with drug delivery staff from TASO to receive drugs in neutral locations. The following quote about the home delivery of drugs with a TASO labelled motorcycle illustrates this point:

… Of course that is not a very comfortable situation (laughs) especially when the motorcycle has something labelled on it like TASO or something like that.

[but now] *the field officer who supplies me usually does not just ride the bike up to my place, he parks it somewhere and then he walks to my place that is if we are to meet at my place, and if not then he tells me to find him somewhere.* (MHH3, 30-month interview)

Echoing the above trend, TASO staff during the group discussions reported a change in attitude towards the visibility of TASO in the participants’ homes. They agreed that some participants in the home based arm were increasingly beginning to show discomfort with the routine drug deliveries yet they had not had any problem with this earlier on. In other cases, some participants had taken on new jobs and were not yet ready to disclose to employers or colleagues at work, yet they had to find time for ART delivery. The participants in the focus group were all in agreement regarding this issue. The following quote captures this perception:

*What I have seen, most of our clients have improved their health, and they have obtained jobs. When they see a TASO counsellor or any provider they always tend to hide. They now do not want to associate with HIV which shows that there is some kind of increased stigma. But before joining and gaining weight when they had all these signs and symptoms, they would even invite you* [to their home]*.* (Male counsellor in FGD of facility based staff)

### Sexual, reproductive health, disclosure and stigma

Perhaps one of the most important aspects of full recovery for participants was the aspect of sexual and reproductive health. There was a strong contrast between the narratives of, for example, many women who as they had begun ART had vowed never to resume sex as they struggled with the various illnesses and sad memories of how they had become infected. The possible negative reactions from a suspicious public were also a deterrent particularly given that many of these people had a very public and visible illness experience either through the death of a spouse or because of their own illness. With time on ART however, by the thirtieth month, seven of these women were in a relationship, had had a child or were currently pregnant. The partners they had were rarely aware of their HIV sero-status and although this worried the women because of the potential for infecting these partners, such decisions not to disclose were often rationally made after weighing up the costs of losing the new relationship. The following excerpt from the interviews illustrates this point:

***Question:****Now, you don’t know his status…how do you feel about the fact that he might contract it* [HIV/AIDS] *if he didn’t have it?*

FHH3***:****Well, the problem is I might tell him and it causes me problems because the ways of men are complicated. He might beat you and say you infected him yet he may at times have it too. Because he is a mature person, it is not me that he first had a relationship with.* (FHH3 30-month interview)

Another woman (FFL2) who had started a relationship with a man whose status she did not know narrated at the 30-month interview that she had ended up getting pregnant shortly after what started as a casual relationship.

***Question:****Did you disclose to him your HIV status?*

FFL2***:****No*

***Question:****But do you think he knows?*

FFL2***:****He doesn’t know* [her status]

***Question:****Do you know about his status?*

FFL2***:****I don’t know*

***Question:****Do you use a condom?*

FFL2***:****We were using, but he reached a time and he lied to me that he had put it on but then I came to realize other things* [She got pregnant]*.*

For the women, increasing age seemed to be associated with avoidance of sexual relationships. Of the eight women who were over 40 at enrolment, only one reported being in a relationship during the 2011 interviews, although this relationship did not involve sexual intimacy because her husband was HIV-negative. She was accepted by her co-wives and as we see in her narrative below, she was happy to maintain her abstinence at age 48 years. She said:

As I have told you that my husband is HIV negative and even my co-wives are HIV negative, we decided not to have sex again and ever since I was found positive, we have never had sex. We have a good relationship with my co-wives and even my husband. I no longer think about those issues. (FHL3, at the 2011 interviews)

In focus group discussions, TASO staff admitted that there was some shame attached to pregnancies which could have arisen from previous messages which were interpreted by clients as discouraging pregnancy. This finding has been reported in more detail elsewhere [23]. A discussant in the focus group had this to say on this topic:

*…You know what happens is that when they* [clients] *come here some especially those ones who are pregnant have the fear or they feel ashamed. You know it’s a process. Usually here such a client has to see a counsellor. So imagine they have been telling a counsellor for some time that they are abstaining and now the counsellor sees that they are pregnant, so if they come here they wonder what they will tell such a counsellor*. (Male, field officer FGD)

Another field officer noted that there was a missed opportunity for further counselling as participants with pregnancies stayed away from TASO. The fear of some staff was that these participants may come to harm because there are some drugs that the providers were made aware of that could harm the unborn baby as well as methods available to reduce the chance of vertical transmission. The participants in the focus group gave examples of this scenario:

*…even me I have one client on combivir/efavirenz, she is now seven months pregnant but whenever I refer her for change of regimen she says that she comes to the clinic but then she disappears without seeing anyone. Now when you go to her home she says that she came and sat there since morning up to 6pm and no one attended to her yet in actual sense her file was retrieved and taken to the clinician but she was being called and she was nowhere to be seen.* (Male field officer FGD)

### Sero-sorting as a strategy against stigma

Some participants deliberately looked for same HIV-positive status partners. This was a strategy to avoid public scrutiny about their sex lives involving people of unknown status or those thought to be uninfected. Although no one experienced any enacted stigma as a result of engaging in a sexual relationship, the reports by those who sero-sorted seemed to point to the possibility of this existing within the community. One female participant observed:

*Let me tell you when there is health, there is life. I tried very much to be single but when I couldn’t hold on any longer I got someone who was also with TASO and is on medication.* (FFL5, 30-month interview)

It is worth noting that those participants, who had started new sexual relationships on the basis of sero-sorting, sometimes depended on the self-report by the new partner. Among the women who had resumed sexual relationships, only one seemed sure of the status of the new partner since they had met at TASO. Another one assumed her partner was HIV positive but never really confirmed this. She tried to make enquiries about him and even encouraged him to visit TASO but he seemed reluctant to do so as seen in the quote below:

*…When he talked about starting a relationship with me I told him about my health status and he also told me that he was HIV positive and on drugs…I asked him to go and see my counsellor and he refused saying that he didn’t have time to go to TASO…I have never seen him taking them* [referring to ART]*… He told me that he takes his drugs at 8am, he goes to work at exactly 7am in the morning and he comes back home at around 10.00pm in the evening. He may be taking them after he has left this home.* (FHL2, 30-month interview)

In some cases a participant either looked for a partner privately from among others within TASO or used friends to link them up to others who were of the same status. The support and wise counsel when it was sought and obtained from TASO staff tended to help them feel comfortable with the choice of partner. The pregnancies that followed new sexual relationships were also easy to deal with when the participants had informed TASO staff, and in such cases advice such as prevention of mother-to-child transmission was given and if any drug changes were necessary, then this was also done. Those who sought help from staff were more confident about their situation than those who hid from TASO. A male participant who obtained a second partner in such a way narrates:

*… A few years ago I found a lady who was HIV positive… then TASO office decided to introduce a community drug distribution point in my community…Whenever I went there I could find her also having gone for drugs. I talked to the person giving out the drugs. He told me that it was okay [to have a baby] if she was also HIV positive. They told us not to breast feed the baby and we did not and already I had some information about PMTCT.* (MFH2, 30-month interview)

### Perceptions on possible re-infection and infectiousness

On the issue of re-infection which was a concern at enrolment and featured heavily in the conversations regarding the adherence counselling, participants seemed aware of the risk but were almost powerless to take action, feeling that having a same status partner was as far as they could go. The following excerpt from an interview with a male participant (MFL4) at the 30-month interviews shows the choices that have to be made by people living with HIV on ART as a strategy for avoiding stigma related to resuming sex, as well as the guilt of possibly infecting others:

***Question:****To what extent does spreading the virus to other people worry you?*

**MFL4*:****It worries me so much and that’s why I got a fellow HIV positive partner because I wanted someone who knew that I have the virus. Now in case I love another person who is not infected, it is like taking a panga* [large knife] *and cutting her. That is real hatred; it is not good to spread this disease.*

***Question:****Now you have said you don’t use condoms, to which extent does contracting other diseases from your partner worry you?*

**MFL4*:****We don’t use it but I know that if she has other diseases she can spread them to me.*

The above participant while demonstrating knowledge about the risk of re-infection in the absence of condoms even after sero-sorting, was still reluctant to use condoms as a means of protection against such risks. This demonstrated how these participants who seemed very knowledgeable had to explore several choices although in some cases the choices were very limited and there was a potential risk in a particular choice taken.

## Discussion

In this paper we have presented the stigma experiences of clients enrolled in an AIDS support organisation from the time they started taking ART over a 6-year period. It is widely agreed now that HIV is a chronic condition which needs to be managed as such and the ammunition to do this is available [32,33]. Our participants had access to a comprehensive package that addresses their psycho-social needs. These people had been deemed to be ready for treatment [17]. For this population as is the case for many people living with HIV the source of stigma were the physical bodily attributes which are significantly discrediting [4,34]. We also noted that infection with HIV in a setting like Uganda requires the affected individual to negotiate various challenges at the personal and structural level. For example individuals have to negotiate various identities from the time of infection in order to fit into the role of an HIV-infected individual [35]. Along the way, individuals may withdraw from activities that might expose them as being infected with HIV [36].

The start of AIDS related bodily symptoms or the fear of these happening in future triggers stigmatizing feelings [12,37]. The inability to conceal symptoms is described as a source of stigma. We found in our study that people develop strategies to overcome the anticipated or real stigma by either withdrawing from public activities as they seek advanced treatment options to cope [17], avoiding disclosure even to sex partners after they have recovered from the debilitating symptoms and resumed their sex lives [21], and sero-sorting which also helps minimize the burden of guilt that one could pass on the infection to someone else [23,38]. However, we also noted that it was not always straight forward. As has been reported elsewhere, sero-sorting may not actually be that protective in situations where the sero-status cannot be established beyond doubt [39,40]. It is worth also noting that sero-sorting may not provide protection against superinfection which could complicate ART treatment or even increase incidence [41,42]. Despite the challenges, some of our participants confronted the stigma bravely having embraced their new illness identity [43]. This latter strategy is the preferred one for the support organisation these people are attached to.

The roll out of ART led to a reported rapid early decrease in stigma among this study population, attributed to the clearing of visible signs in the majority of the cases. However new dilemmas arise as health improves. The need to restart or fit into expected societal roles may drive behaviour [33]. As health improves and people resume their lives and livelihoods, stigma associated with drug taking may emerge. Some participants in our study preferred not to disclose their status arguing that it served their interests better by keeping this secret. The reluctance to disclose to new employers/sexual partners and continue unprotected sex with such partners despite thorough counselling from TASO further illustrates this dilemma. Resumption of sexual relationships ties in with the cultural pressure to have children, which is a cherished goal of many women in countries such as Uganda and it appears that improvements on ART seems to be resurrecting this desire [44].

This study presents interesting developments about the changes in stigma among people living with HIV on ART over a period of about three years and some insights into their lives after the close of the 2005–2008 study. However, it has some limitations which need to be taken into consideration when interpreting these results. This study was conducted among TASO clients who receive good regular counselling and their high rates of adherence and reduced levels of stigma could be as a result of the psycho social support they receive. This makes it hard to generalize these results to populations that do not access this professional help. The possibility that others may stay away from TASO could potentially be attributed to stigma.

Nevertheless, the changes in social and livelihood options that we began to notice towards the end of 30 months and the associated stigma that was unanticipated and never captured in the psycho social readiness activities at enrolment seem to cause challenges which may eventually affect high long term adherence as well as having implications for prevention and care.

## Conclusions

Pre-ART stigma was reportedly high in this population manifested in withdrawal from social activities to avoid blame and shame. HIV-related stigma is reported to have reduced after initiation of ART but as time passed conditions changed, new situations were encountered, and this may have triggered an increase in the stigma participants taking ART felt. The illness and dependence on others that these participants report before starting ART motivated them to adhere well. The dramatic positive changes that occurred after initiating ART helped reduce stigma. However, as lives were rebuilt and the social and economic contexts began to change, there was a noticeable re-emergence of stigma which began to take shape as PLHIV restarted social, economic and emotional roles. This stigma was related to bodily changes and in some cases being seen taking ART. They tried to recreate a new identity which tended to downplay the importance of their HIV infection for fear of destabilizing their newly acquired identities. Sero-sorting also gained prominence as a strategy to manage infection and new relationships. Adherence to drugs may become challenging, which calls for the development of new strategies by support organisations to deal with this new face of stigma. We recommend that innovative counselling messages be reinforced periodically and that an appreciation of the changing patterns of stigma be reflected in these messages.

## Competing interests

The authors declare that they have no competing interests.

## Authors’ contributions

MM participated in the design of the study. He also coordinated the social science component of the trial, carried out the analysis and wrote the first draft of the manuscript and revised the manuscript. JS provided technical advice on the study design, analysis and interpretation of data and helped write the paper. RK contributed to the analysis and interpretation of the data. SN participated in data collection, analysis and writing of sections of the manuscript. JB coordinated the ART programme at TASO Jinja and reviewed the manuscript, SJ was the principal investigator of the Jinja trial, was involved in the design of this study and he critically reviewed this manuscript. All authors read and approved the final manuscript.

## Pre-publication history

The pre-publication history for this paper can be accessed here:

http://www.biomedcentral.com/1471-2458/13/804/prepub
